# Near-field visualization of plasmonic lenses: an overall analysis of characterization errors

**DOI:** 10.3762/bjnano.6.211

**Published:** 2015-10-26

**Authors:** Jing Wang, Yongqi Fu, Zongwei Xu, Fengzhou Fang

**Affiliations:** 1 School of Physical Electronics, University of Electronic Science and Technology of China, Chengdu 610054, Sichuan Province, P. R. China; 2 Key Laboratory of Precision Measuring Technology & Instruments, Centre of MicroNano Manufacturing Technology, Tianjin University, 300072, P. R. China

**Keywords:** characterization, nanofabrication, near-field, plasmonic lenses, plasmonic structures

## Abstract

Many factors influence the near-field visualization of plasmonic structures that are based on perforated elliptical slits. Here, characterization errors are experimentally analyzed in detail from both fabrication and measurement points of view. Some issues such as geometrical parameter, probe–sample surface interaction, misalignment, stigmation, and internal stress, have influence on the final near-field probing results. In comparison to the theoretical ideal case of near-field probing of the structures, numerical calculation is carried out on the basis of a finite-difference and time-domain (FDTD) algorithm so as to support the error analyses. The analyses performed on the basis of both theoretical calculation and experimental probing can provide a helpful reference for the researchers probing their plasmonic structures and nanophotonic devices.

## Introduction

The characteristics of nanophotonic devices that are based on surface plasmon polaritons (SPPs) are appealing because of the extraordinary transmission in free space [1–6]. Many SPP-based superlenses were presented recently [7–9] as well as resonance immunosensors [10], nanobiosensors [11] and nanoprobes [12]. One of the superlenses consists of a silver film coating on glass with some corrugations [13]. Meanwhile, similar results were reported by other researchers [14–17]. They claimed that breaking the diffraction limit is possible by using plasmonic structures [18–20]. However, the shape of the focal point is not ideal. In order to achieve a focused spot with circular shape, we designed the plasmonic structures consisting of elliptical slits [21]. This plasmonic lens has been selected here as a typical example for the purpose of illustrating and analyzing the characterization errors originated from the nanofabrication process of the plasmonic lenses.

**Figure 1:**
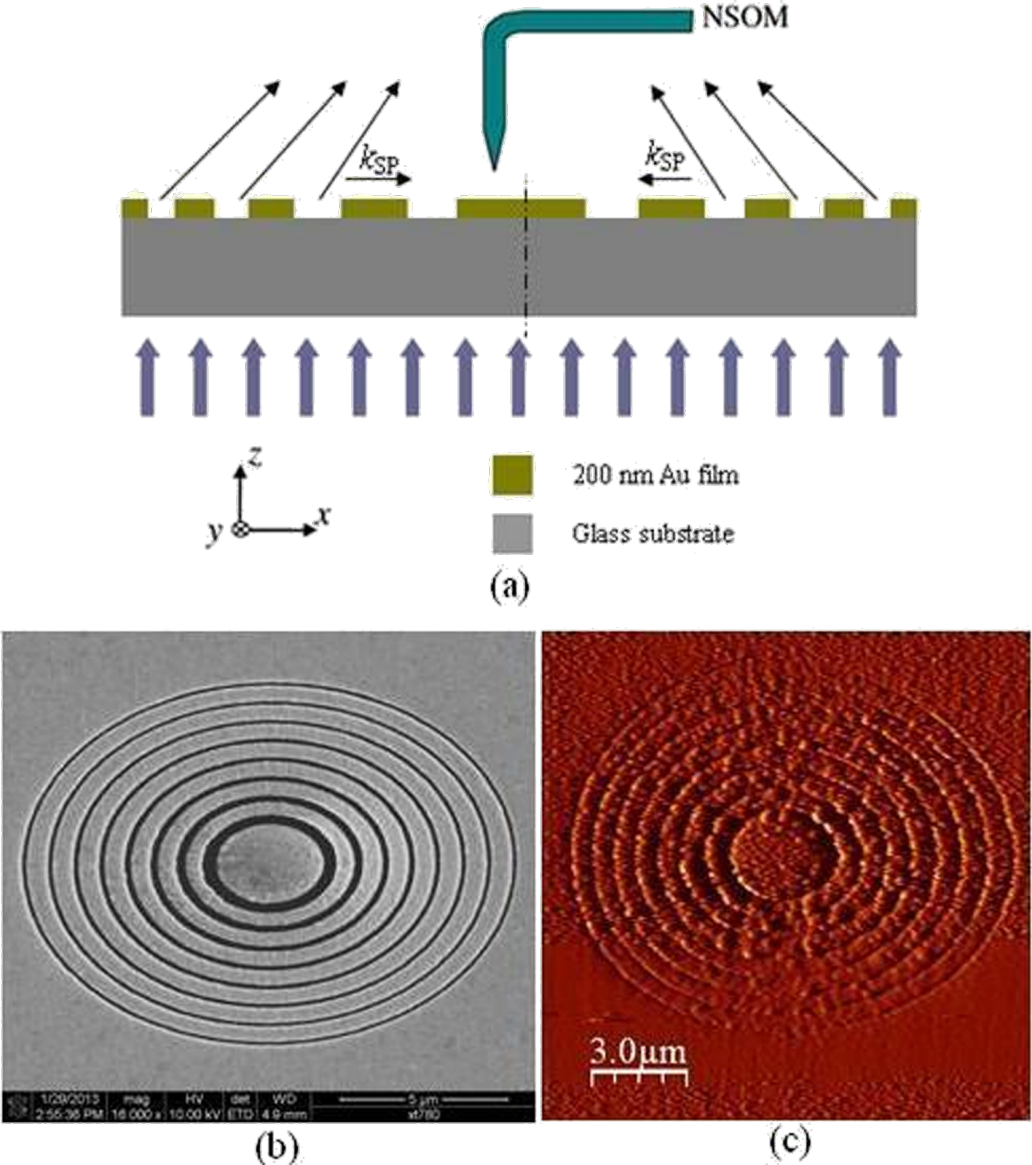
(a) Schematic diagram of the plasmonic structures for focusing based on elliptical slits. Orientation of the long axis of the elliptical slits is in *x*-direction. (b) SEM image of fabrication pattern using FIB. (c) NSOM probed phase map of the plasmonic structure with a ratio of σ = 0.8.

The focused spot can be tuned by means of tailoring the long and the short axes of the elliptical slits. The focusing performance of the structures was studied before [22]. The structures can be fabricated and measured by using focused ion beam (FIB) direct writing technique and near-field scanning optical microscope (NSOM) respectively, as shown in Figure 1.

However, from the point of view of optical nanoscopy, it is a challenge to accurately measure the plasmonic structures at near-field due to characterization errors that originate from both fabrication and near-field probing processes. In this paper, an overall analysis of all the factors that influence the characterization of plasmonic structures, such as lensing structures based on elliptical slits, is presented and discussed in detail.

## Experimental

The FIB milling and NSOM experiments were performed in a similar manner as described in [23]. To illuminate the lenses uniformly, sample scan is used in the near-field mapping. The aperture size of the fiber probe being used in this experiment is 200 nm in diameter. As a consequence, image resolution of the NSOM scanning is limited especially for probing the topography of the FIB-fabricated structure. But the phase image is still clear, as shown in Figure 1c. Considering the radius of the lens and resolution limits, the scanning region was defined as 17 × 17 μm^2^ at 256 points per scan line. Both tip scan mode and sample scan (moving stage) modes were used to probe the lens.

## Results and Discussion

**Figure 2:**
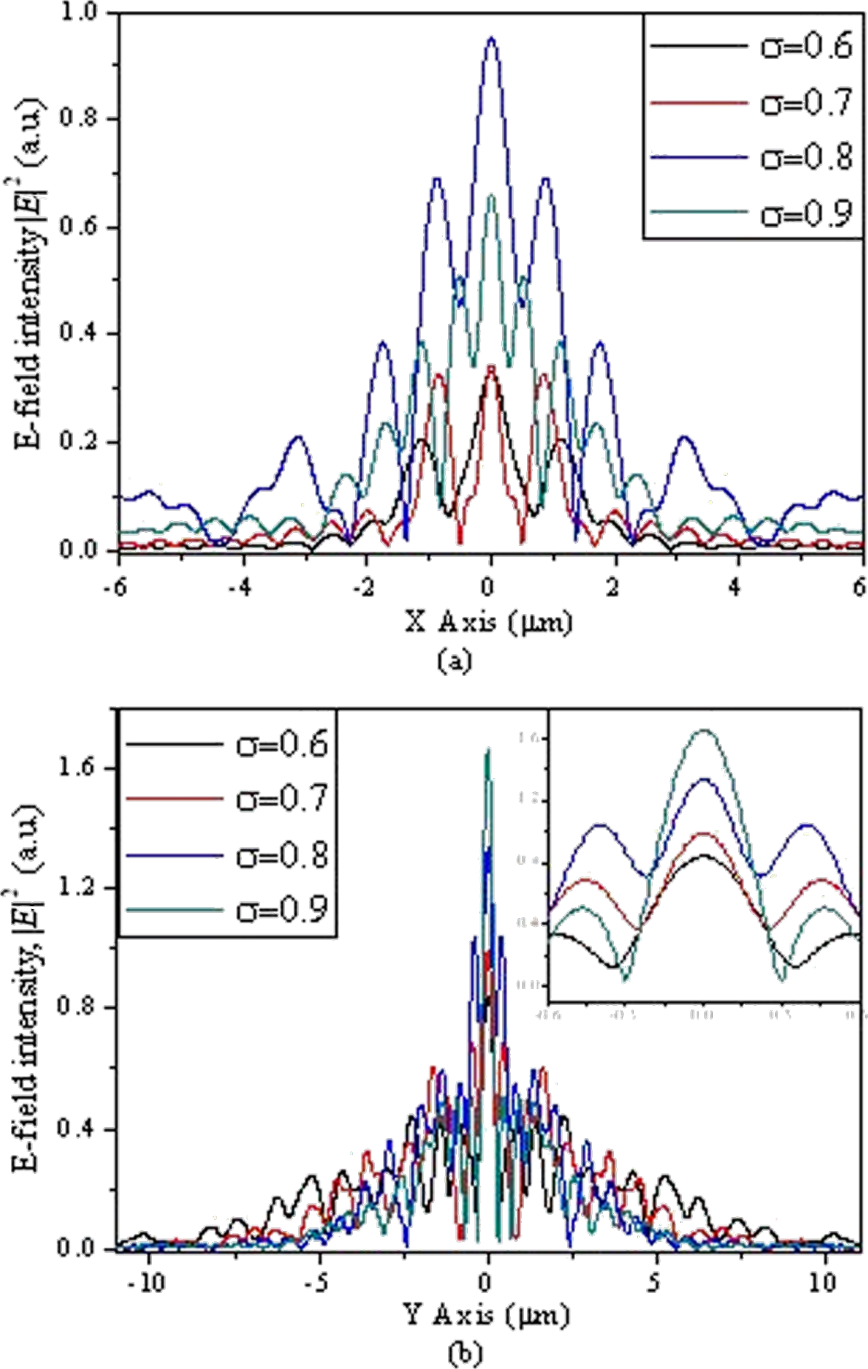
Calculated profiles of the *E*-field intensity |*E*|^2^ in *x*-direction for structures with different ratios σ in (a) *x*-polarization and (b) *y*-polarization. The insets show a zoom-in of the central peaks.

**Figure 3:**
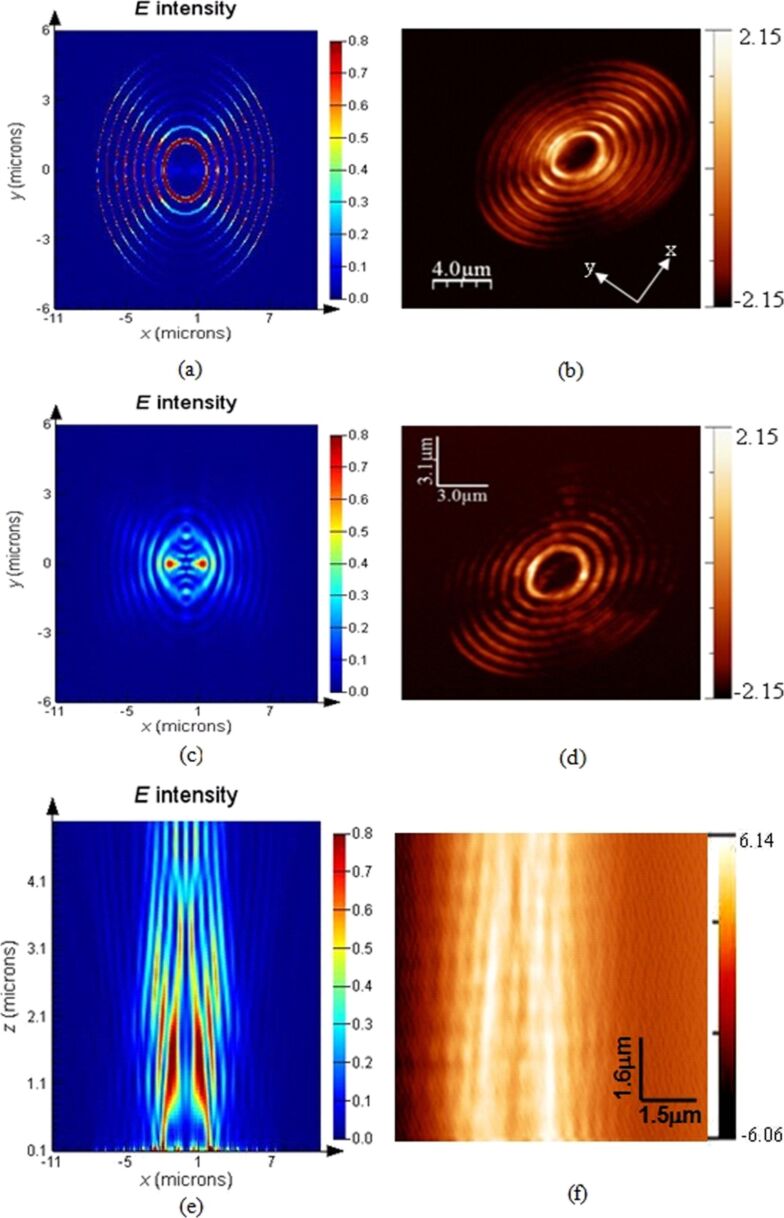
Comparison of *E*-field intensity distribution under *x*-polarization between calculation and NSOM measurement of the plasmonic structure with a ratio σ = 0.7 probed at the position of (a) calculation, *z* = 10 nm; (b) NSOM-probed, *z* = 10 nm; (c) calculation, *z* = 1 μm; (d) NSOM-probed, *z* = 1 μm; (e) calculated *E*-field intensity distribution in the *x*–*z* plane; (f) NSOM probed *E*-field intensity distribution in the *x*–*z* plane.

**Figure 4:**
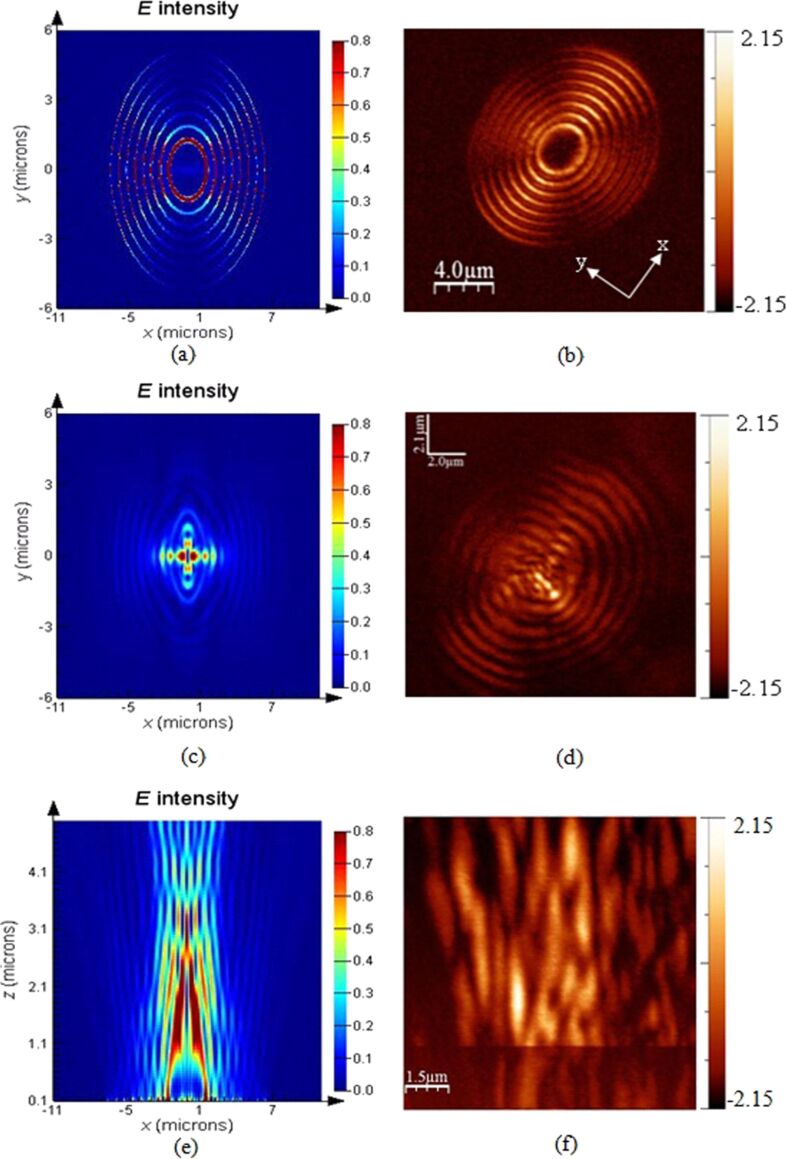
Comparison of *E*-field intensity distribution under *x*-polarization between calculation and NSOM measurement of the plasmonic structure with a ratio σ = 0.8 probed at the position of (a) calculation, *z* = 10 nm; (b) NSOM-probed, *z* = 10 nm; (c) calculation, *z* = 2 μm; (d) NSOM-probed, *z* = 2 μm; (e) calculated *E*-field intensity distribution in the *x*–*z* plane; (f) NSOM probed *E*-field intensity distribution in the *x*–*z* plane.

**Figure 5:**
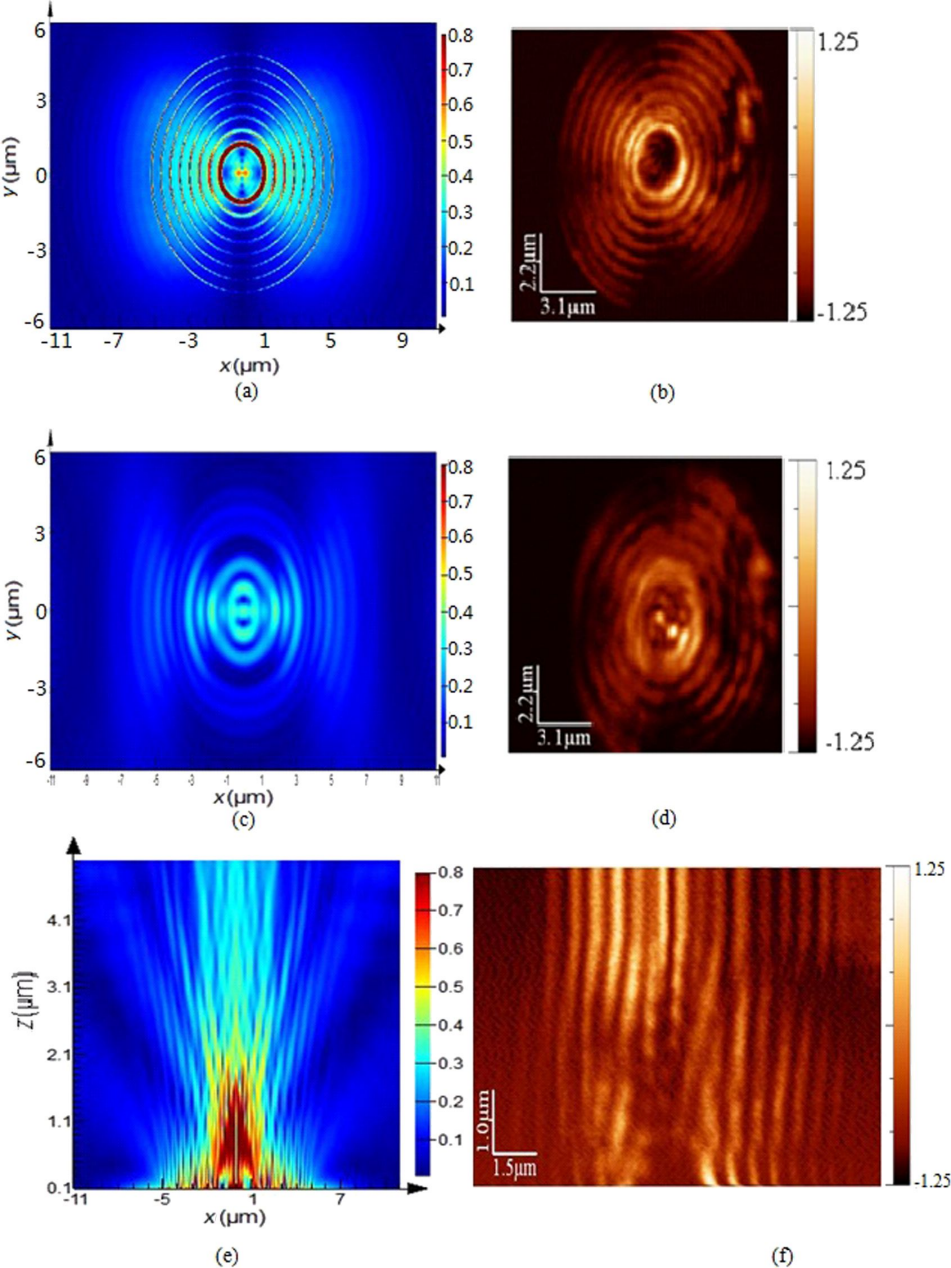
Comparison of *E*-field intensity distribution under *x*-polarization between calculation and NSOM measurement of the plasmonic structure with a ratio σ = 0.9 probed at the position of (a) calculation, *z* = 10 nm; (b) NSOM-probed, *z* = 10 nm; (c) calculation, *z* = 3 μm; (d) NSOM-probed, *z* = 3 μm; (e) calculated *E*-field intensity distribution in the *x*–*z* plane; (f) NSOM probed *E*-field intensity distribution in the *x*–*z* plane.

**Figure 6:**
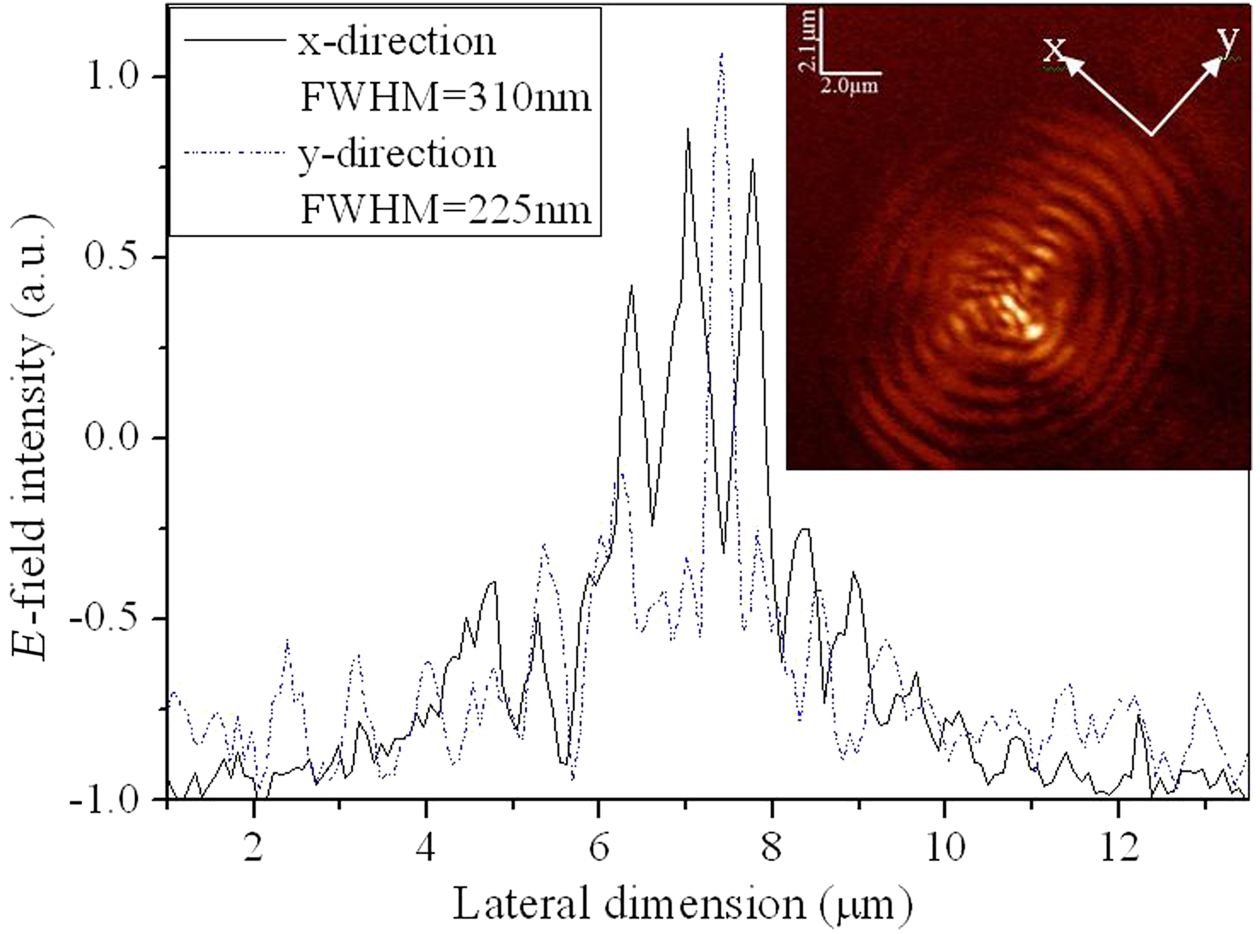
NSOM-measured *E*-field intensity profiles along *x*- and *y*-directions for the case of σ = 0.8 at *z* = 3 μm.

The plasmonic structures based on elliptical slits can finely focus after the exit plane in free space. The perforated elliptical slits are adopted here for the purpose of controlling the focused region from both *x*- and *y*-directions. The focal region is formed by the SPPs-enhanced interference of the diffraction wavelets originating from the slits [5,13]. Figure 2 shows the calculated intensity profiles of the electric field for plasmonic lenses with different ratios σ under plane wave illumination. The working wavelength of the lenses is 532 nm. Three dimensional (3D) calculations were carried out on the basis of finite-difference and time-domain (FDTD) algorithm. The elliptical ratio is defined as σ = *a*/*b*, where *a* and *b* are the short axis and the long axis, respectively. It can be seen that the focusing performance is best, with a high peak intensity and a small spot size at site of full-width and half-maximum (FWHM), for σ = 0.8. Theoretically, FWHM can be beyond the diffraction limit (less than half of the incident wavelength of 532 nm). To verify it experimentally, FIB and NSOM were employed for nanofabrication and near-field characterization, respectively. Figure 3, Figure 4 and Figure 5 are NSOM probing results for the lenses with different ratios σ ranging from 0.7 to 0.9. Figure 6 shows the probed two-dimensional (2D) *E*-field intensity |*E*|^2^ profiles in *x*- and *y*-directions for the case of σ = 0.8 and *z* = 3 μm. As can be seen, the measured FWHM of 225 nm (this measured value involves some of the analyzed characterization errors presented below, especially the influence of probe interaction and misalignment) in *y*-direction is beyond the diffraction limit. It is in agreement with the theoretical calculation results despite the existence of optical nanoscopic errors from both fabrication and characterization.

### Optical nanoscopic errors

#### Elliptical ratio σ

**Figure 7:**
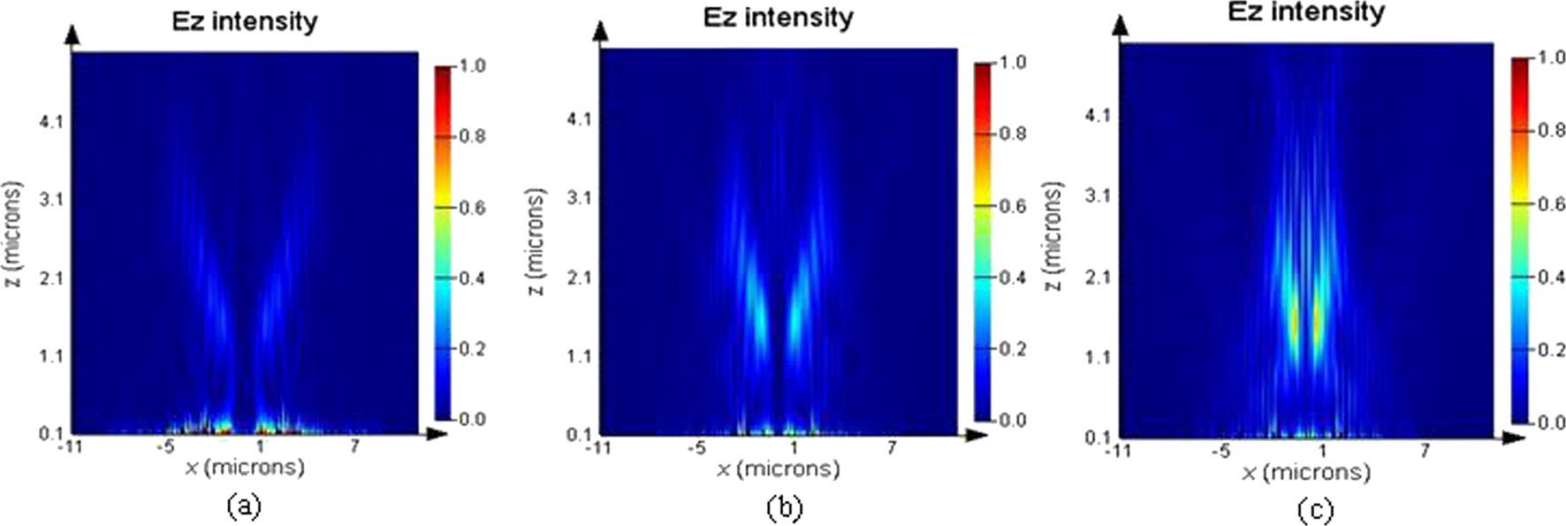
Intensity distribution of the *E*·*x* component in the *x*–*z* plane under *x*-polarization for (a) σ = 0.7; (b) σ = 0.8; and (c) σ = 0.9, respectively.

The elliptical ratio of the plasmonic structures determines focal spot size, region, and depth of focus. In order to excite SPP waves for enhanced transmission, p-polarization is employed. Our calculation results demonstrate that no focal region can be formed in free space due to the destructive interference caused by the large long axis under illumination of linear polarization if σ ≤ 0.5. From the point of view of near-field mapping, the signal collected by the fiber probe is |*E*·*z*|^2^ instead of the total *E*-field intensity |*E*|^2^. The apertured NSOM probe is more sensitive to 
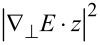
 and the detected signal of the NSOM fiber probe is proportional to 
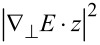
 [24–25]. Only the longitudinal field component *E*·*z* can be collected by the probe and the transverse component *E*·*x* does not enter into the probe. Therefore, theoretically, the intensity of the near-field images is lower than that of the calculation. However, the transverse component *E*·*x* makes a positive contribution to width and density of the interference fringes. No collection of *E*·*x* by the probe means that the fidelity of the probed fringes cannot be guaranteed. As can be seen from Figure 4f and Figure 5f, both the fringe density and width vary in comparison to the calculated image shown in Figure 4e and Figure 5e. Apparently, the measured width is enlarged and the density is reduced due to absence of a contribution *E*·*x* during the probing process. In contrast, we give the intensity distribution of the *E*·*x* component in the *x*–*z* plane under *x*-polarization for the cases of σ = 0.7, 0.8 and 0.9 shown in Figure 7.

#### Probing interaction

The interaction and perturbation of evanescent fields between a facet of a metal-coated fiber probe and the surface of a plasmonic structure will influence the final probed NSOM image in the near field. A previous study demonstrated that for the case of 10 < *h* < 30 nm, the measured optical field is largely different from the original *E*-field without the probe interaction, especially for the sharp peak value. The measured field distribution will accurately depict the original distribution when 40 < *h* < 80 nm [26]. The probe is far from the plasmonic lenses and can detect only a small amount of the near field component in the case of *h* > 80 nm. In addition, the interaction and perturbation only affects the electric field intensity, and there is no influence on the phase distribution of the plasmonic structures. To avoid this influence, the fiber probe without metal coating at the tapered section can be used as long as the optical energy loss at the tapered section is acceptable for imaging with apertured NSOM. In addition, the cone angle of the fiber probe will cause a geometrical characterization error when probing slits with nanoscale dimensions [27]. In this case, measurement errors in both the vertical and horizontal directions of the nanoscopic slits can even be doubled.

#### Misalignment of fiber probe

The influence of misalignment of the fiber probe on near-field mapping is apparent for the symmetrical plasmonic structures and nanophotonic devices having a central axis. In this case, it is necessary to adjust the alignment between the fiber probe and optical axis of the illumination system. Misalignment of the probe will cause offset of the focused spot/region of the lenses, as shown in Figure 4d and Figure 4f, which are NSOM images for the case of σ = 0.8, *z* = 2 μm. It can be seen from Figure 4d that the focal spot is apparently offset in comparison to the calculated image (see Figure 4c). The region with high intensity in Figure 4f marked “A” represents the influence of the misalignment. Similarly, the images shown in Figure 5d and Figure 5f for the case of σ = 0.9, *z* = 3 μm also verify the influence.

In addition, a slanted sample surface due to improperly fixing the sample on the stage or the flatness of the sample itself will generate the phenomenon. Offset of the optical axis is *h*·tanθ, where *h* is the total thickness of the sample, and θ is the tilt angle of the sample.

### FIB nanofabrication error

#### Stigmation

**Figure 8:**
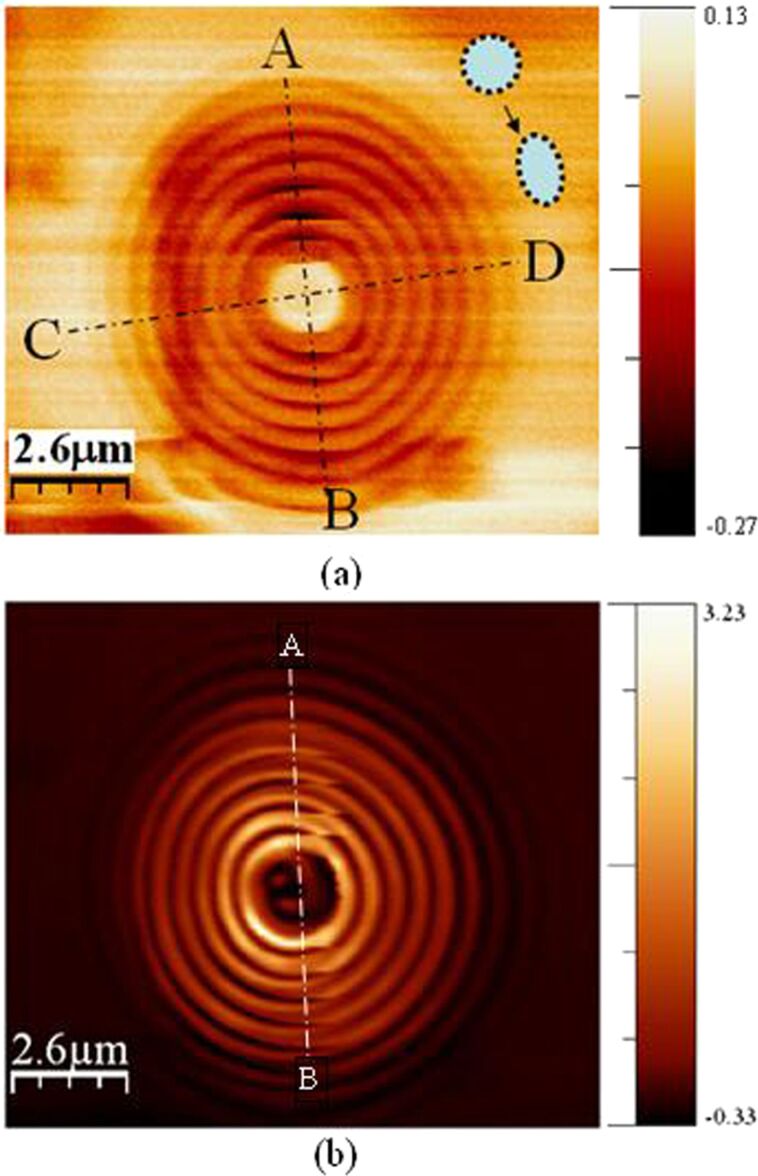
Influence of stigmation of FIB for the special case of σ = 1 (circular slits). (a) AFM probed topography of the structure; (b) corresponding NSOM measurement result.

Stigmation is generated in the ion column of the FIB machine due to asymmetrical voltage applied on the stigmator consisting of octopole electrodes. The ion beam is distorted due to the asymmetrical voltage distribution on the electrodes. The energy spreading is asymmetrical and produces an elliptical spot of the ion beam instead of the normal circular spot. Theoretically, it can be expressed as Equation 1 [28]:

[1]
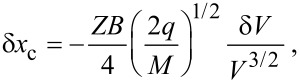


where *B* is the magnetic field, *q* is the velocity of an ion, *M* is the mass, *V* is the accelerating voltage, and *Z* is the mass selection size. In practice, this is sufficiently small to be removed by astigmators located downstream of the filter. The stigmation strongly depends on the fluctuation of voltage which generates the voltage variation δ*V*. Theoretically, it will be sufficiently small as long as the stability of the voltage is high enough. Normally, the voltage fluctuation is controlled at a variation level of ±0.01%. Elliptical ion beam spot instead of circular spot will produced due to stigmation. The elliptical ion beam spot causes a deformation of the fabricated pattern, as shown in Figure 8. A lens with σ = 1 (circular slits) was milled using FIB direct writing. But the fabricated structure is deformed along the long (as indicated by A–B line) and the short axis (as indicated by C–D line) due to stigmation during patterning, as shown in Figure 8a. The dotted circular and elliptical shapes represent the ion beam spot focused in ideal case and the case of stigmation, respectively. Then the deformed structure is probed at near-field using NSOM, as shown in Figure 8b. It can be seen that the deformed slits lead to dislocation of the near-field intensity distribution along the A–B line. For FIB patterning, the inherent astigmatism will exist to a certain extent no matter how finely the operator calibrates the stigmation. Stigmation causes excessive overlapping of the focused ion beam spot along long-axis. Sometime, the overlap can be as large as 90% which is obviously too large for the FIB point-by-point writing.

#### Internal stress

FIB bombardment with ion energies below 30 keV produces surface diffusion and tension down to about 30 nm underneath the surface of the etched structures. One by-product of ion beam sputtering is erosion. The arrival of ions on the sample surface is a stochastic process which causes the roughened top surface of the sample. However, the roughening process is a dynamically instable state and competes with surface smoothing process. Therefore, internal stress is generated when a balanced state is reached, unless thermal annealing is arranged after the patterning. However, oxidation of the metal film will be caused by thermal annealing. The internal stress can lead to deformation of the fabricated structures and the NSOM measurement results will change accordingly. The internal stress τ is determined by the following parameters

[2]
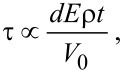


where *d* is the ion implantation depth, *E* is the bombardment energy (30 keV here), ρ is the density of the substrate material, *t* is the milling time, and *V*_0_ is the ion distribution volume originating from the implantation. It can be seen that τ is proportional to implantation depth, ion energy, milling time and material density. For the fixed process parameters, selecting the a substrate materials with small ρ can reduce the internal stress. Theoretically, thermal annealing or natural annealing is necessary after FIB structuring for the purpose of eliminating the internal stress. With regard the quality of the results thermal annealing is better than natural annealing. Due to experimental limitations natural annealing was carried out for our sample before the characterization.

Besides the above mentioned systematic errors, some random errors also exist during the probing, e.g., interference of electromagnetic noise (such as cellphone, radio, and other RF antenna devices) from neighbored areas, fluctuations of the ambient temperature, aging of the fiber probes, and fluctuation of the laser power.

## Conclusion

In summary, characterization errors for near-field mapping of plasmonic lenses based on elliptical slits are analyzed from an optical nanoscopic point of view. Some factors such as elliptical ratio, probe interaction, misalignment, stigmation, and internal stress, are discussed in detail. Significant probing errors originating from both fabrication and characterization can be directly produced or indirectly induced. Probe interaction commonly existed for all the plasmonic structures. The probe interaction perturbation can be avoided by using a fiber probe without metal coating if applicable. For the measured samples without metal surface, contribution from this issue is only little. Misalignment generated probing error only exists for mapping symmetrical structures with a central axis. But the influence of inherent stigmation and internal stress originating from the FIB processing on the accurately probing is too difficult to be eliminated completely unless another nanofabrication technique, e.g., e-beam direct lithography, or nano-photolithography is employed. Especially for the stigmation, it strongly depends on the experience and the skill of the FIB operators.
